# Needle arthroscopy in anatomical reconstruction of the lateral ankle: a report of three cases with a parallel comparison to the standard arthroscopy procedure

**DOI:** 10.1186/s40634-022-00510-x

**Published:** 2022-07-30

**Authors:** R. Lopes, T. Noailles, G. Padiolleau, N. Bouguennec, T. D. Vieira

**Affiliations:** 1 Pied Cheville Nantes Atlantique, Clinique Brétéché 3 rue de la Béraudière, 44000 Nantes, France; 2 Pied Cheville Nantes Atlantique, Santé Atlantique, Avenue Claude Bernard, 44800 Saint-Herblain, France; 3 Département de Chirurgie Orthopédique, Polyclinique de Bordeaux Nord, 15, rue Claude-Boucher, 33000 Bordeaux, France; 4 Clinique du Sport de Bordeaux-Mérignac, 2 rue Negrevergne, 33700 Merignac, France; 5 Centre Orthopédique Santy, FIFA Medical Center of Excellence, Groupe Ramsay-Generale de Santé, 24 Avenue Paul Santy, 69008 Lyon, France

**Keywords:** Ankle reconstruction, Ankle arthroscopy, Needle arthroscopy, Ankle ligaments, Arthroscopy, Nanoscope

## Abstract

**Purpose:**

This study evaluates the use of the needle arthroscopy in anatomical reconstruction of the lateral ankle. We hypothesized that the needle arthroscopy would allow anatomical reconstruction to be performed under arthroscopy.

**Methods:**

Three patients underwent treatment of chronic ankle instability. The comparative procedure was performed in the following four steps: 1) anteromedial articular exploration (medial/lateral gutter/anterior chamber/syndesmosis); 2)creation of the talar tunnel via the anteromedial arthroscopic approach; 3) anterolateral fibular tunneling; and 4) positioning of the graft by the anteromedial arthroscopic approach.

For each of these steps, the planned procedure using the needle arthroscope was compared to the standard arthroscope. For each step, the planned procedure using the needle arthroscopy was compared to the standard arthroscope and the act was classified based on level of difficulty: facilitated, similar, complicated and impossible.

**Results:**

The exploration of the medial and lateral gutter, the creation of the tunnel of the talus and graft positioning were not accomplished using the needle arthroscope. While the syndesmosis visualization was facilitated by the needle arthroscope in comparison to the standard arthroscope.

**Conclusion:**

The anatomical reconstruction of the lateral ankle, using the needle arthroscopy-only approach, was impossible in all three cases, regarding: ankle joint exploration, creation of the tunnel of the talus and graft positioning. The needle arthroscope should not be considered as a "mini arthroscope" but as a new tool with which it is necessary to rethink procedures to take advantage of the benefits of this instrument.

## Case presentation

Three patients underwent surgery for the treatment of chronic lateral ankle instability.

The demographic data of the patients are presented in Table 1.

**Table 1:** Demographic data

		**Patient 1**	**Patient 2**	**Patient 3**
Patient	Sex	Male	Male	Female
	Age	22	36	24
	Side	Right	Right	Left
	BMI (kg/m2)	24	21	20
	Symptoms	Pure instability	Pure instability	Painful instability
	Sports level	Competition	Recreational	None
Intervention	Associated lesions	None	Synovitis Ossification under lateral malleolar	Synovitis
	Tourniquet time (minutes)	47	50	49

**Fig. 1:**
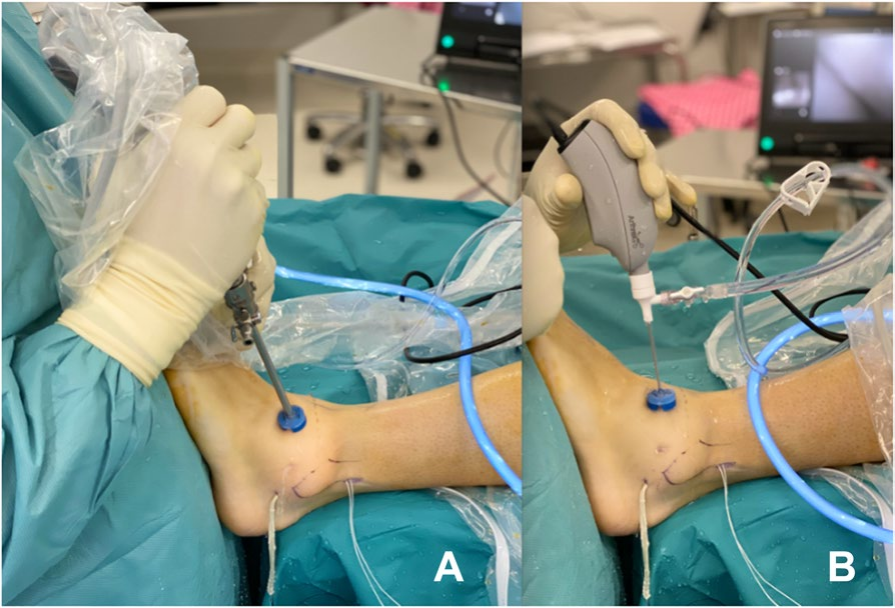
Standard arthroscope being replaced by the NanoScopeTM. **A** Standard arthroscope in the anterolateral portal. **B** NanoScopeTM in the anterolateral portal

The procedure was performed according to a previously described technique [13] and based on previously cadaveric study [14] under general anesthesia. In the light of minimally invasive surgery, less tissue damage and consequently better recovery, the same anatomical reconstruction technique was tested with needle-arthroscopy. During this procedure, the standard arthroscope was sequentially replaced by the needle arthroscopy (NanoScopeTM, Arthrex, Naples, FL) (Fig. 1).

**Fig. 2:**
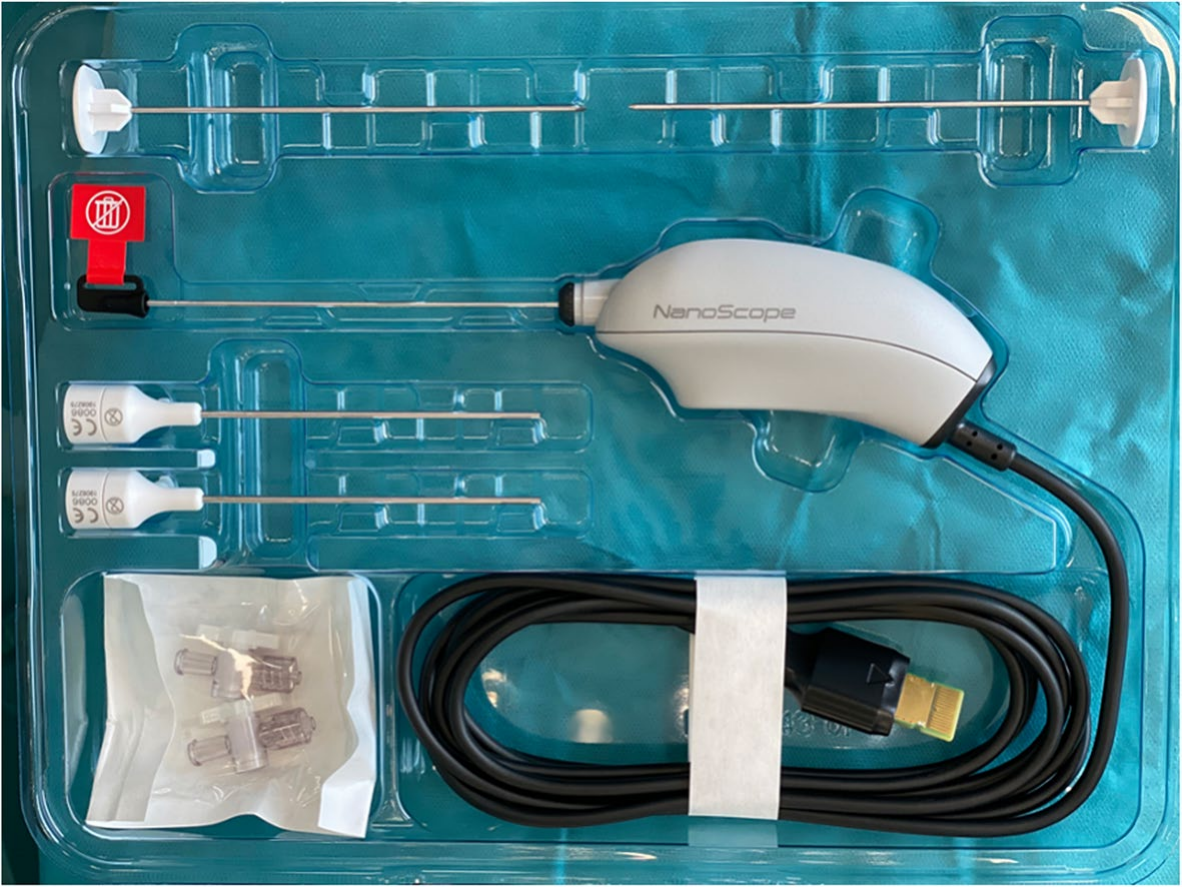
NanoScopeTM video system: handpiece provided in a single-use kit

**Fig. 3:**
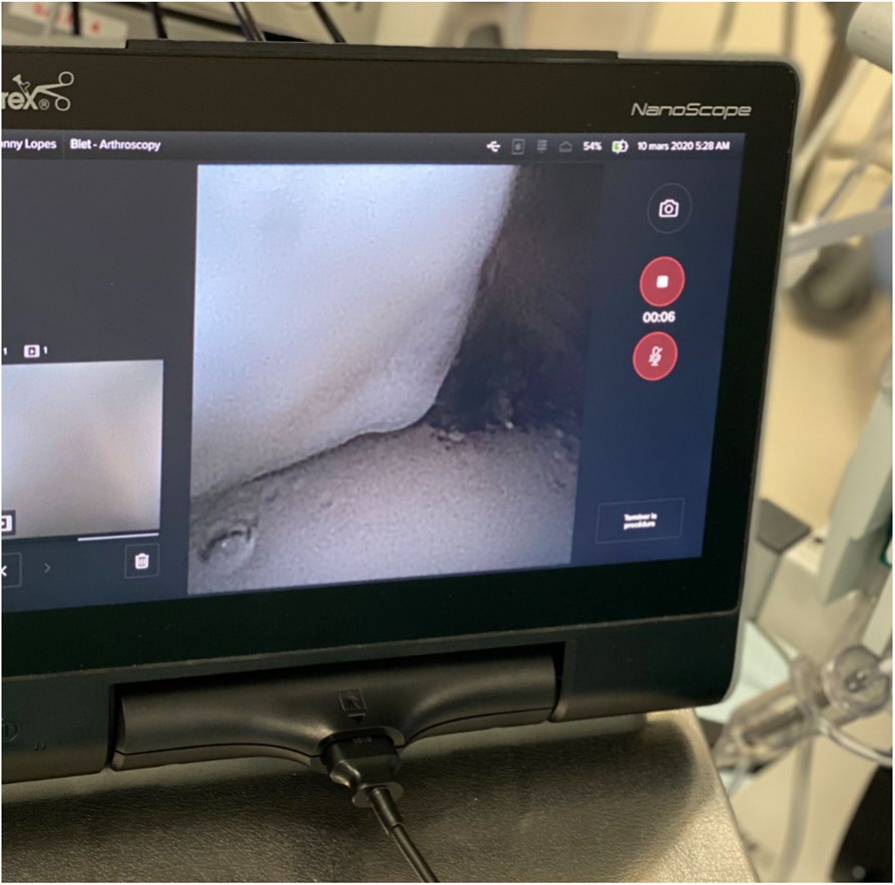
NanoScopeTM 13-inch handheld console

The needle arthroscopy is a video system consisting of a handpiece provided in a single-use kit (Fig. 2) connected to a 13-inch handheld console (Fig. 3).

The handpiece includes an LED light and video capture system. Inserted in a 2.2-mm trocar connected to an irrigation system, its tip has no obliquity and offers a 120° viewing angle. The needle arthroscope is 4.1 cm wide and 25.4 cm long, weighs 150 g and offers an image resolution of 400 × 400 pixels. The overall characteristics of the needle arthroscope are compared with those of the standard arthroscope used during the procedure in Table 2.

**Table 2:** Comparative technical characteristics of the 2 scopes used

	Arthroscope	NanoScope™
Handpiece width	4.5 cm	4.1 cm
Handpiece length	10 cm	25.4 cm
Handpiece height	4.5 cm	6.4 cm
Handpiece weight	570 g	150 g
Diameter without a cannula	4 mm	1.9 mm
Diameter with a cannula	4.6 mm	2.2 mm
Image resolution	1920 × 1080 pixels	400 × 400 pixels
Frame rates	NA	30 ips
Fields of vision	100 degrees	120 degrees
Viewing Angle	30 degrees	0 degrees
Enlightenment	1.800 lm	≥ 2 lumens
Screen size	32 inches	13 inches

The comparative procedure was performed in the following four steps:

1. anteromedial articular exploration (medial/lateral gutter/anterior chamber/syndesmosis) (supplementary video) [5, 11, 24];
2. creation of the talar tunnel via the anteromedial arthroscopic approach;
3. anterolateral fibular tunneling; and
4. positioning of the graft by the anteromedial arthroscopic approach.

For each of these steps, the planned procedure using the needle arthroscope was compared to the standard arthroscope, and the act was classified based on level of difficulty: facilitated, similar, complicated and impossible. The 4 possible task difficulty levels were predefined by the senior author. This comparison is presented in Table 3.

**Table 3:** Interest in using the NanoScopeTM at different stages

		**Patient 1**	**Patient 2**	**Patient 3**
**Exploration**	*Medial gutter*	Complicated	Complicated	Complicated
	*Anterior chamber*	Similar	Similar	Similar
	*Lateral gutter*	Impossible	Impossible	Complicated
	*Syndesmosis*	Facilitated	Facilitated	Facilitated
**Creation of the tunnel of the Talus**		Complicated	Impossible	Impossible
**Creation of the fibular tunnel**		Similar	Similar	Similar
**Graft positioning**		Complicated	Complicated	Complicated

The exploration of the anterior chamber is presented in the video (supplementary material).

**Fig. 4:**
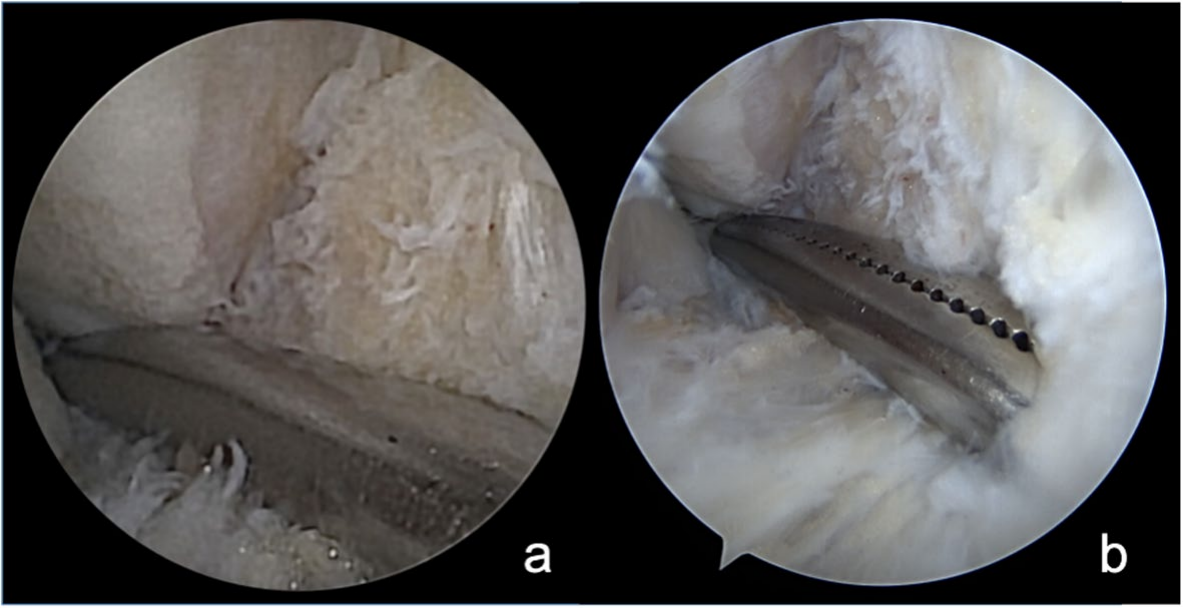
Halstead forceps in the lateral talar gutter. **A** NanoScopeTM view. **B** Standard arthroscope view

**Fig. 5:**
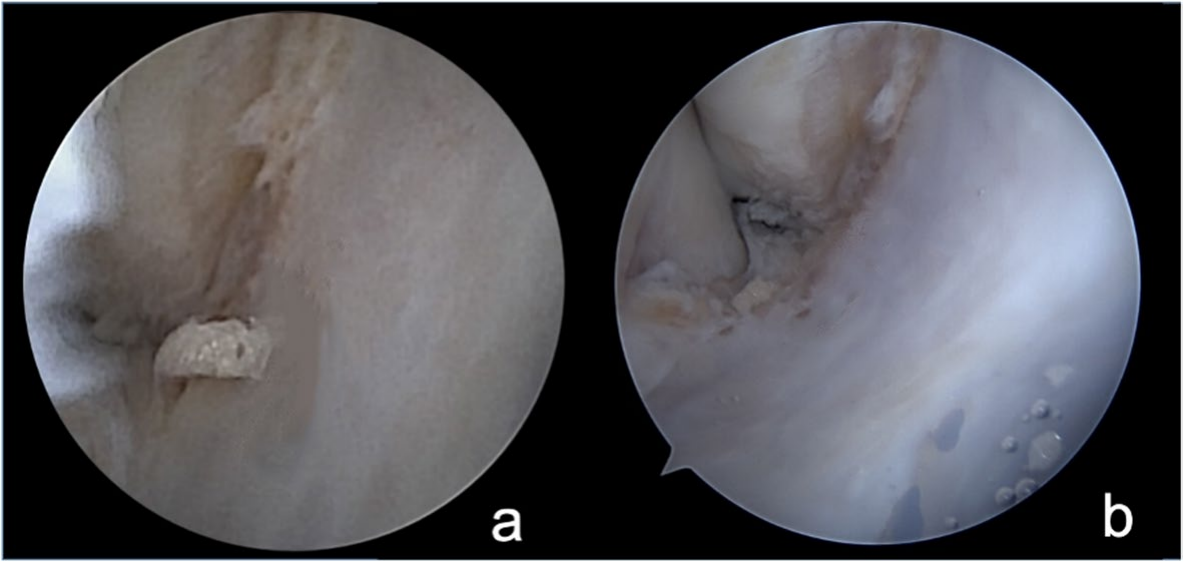
Placement of the cannula in the lateral gutter. **A** NanoScopeTM view. **B** Standard arthroscope view

**Fig. 6:**
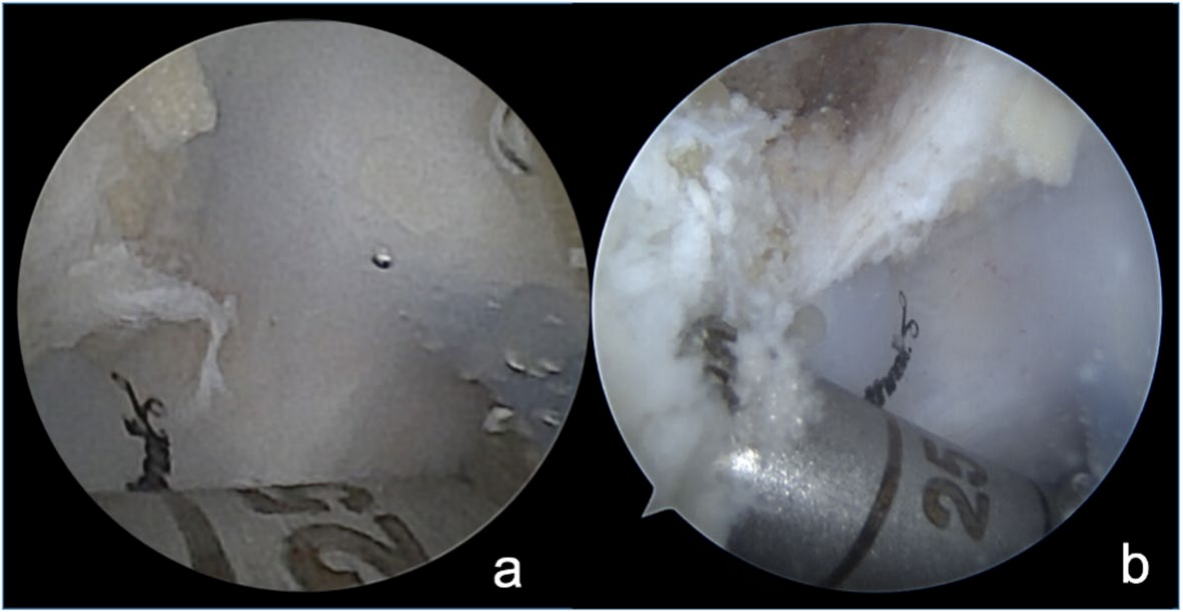
Talar tunnel drilling. **A** NanoScopeTM view. **B** Standard arthroscope view

The view of the lateral talar gutter at different times during the procedure was compared with the 2 instruments (Figs. 4, 5, and 6).

The exploration of the medial and lateral gutter, the creation of the tunnel of the talus and graft positioning were not accomplished using the needle arthroscope. While the syndesmosis visualization was facilitated by the needle arthroscope in comparison to the standard arthroscope.

## Discussion

The main finding of this study was that the needle arthroscopy-only approach did not allow the performance of the anatomical reconstruction of the lateral ankle in the three cases evaluated. This is the first is the first non-cadaveric study that compares step-by-step the surgery with the needle arthroscopy to standard ankle arthroscopy. In the present study, the authors evaluated only the feasibility and not other aspects of interest compared to a standard arthroscope. Overall, the perceived advantages of the needle arthroscope in this study were: ergonomics, smaller diameter, and optics flexibility, which could lead to less iatrogenic risk, and the limitations were: absence of obliquity and low resolution.

Despite the innovative evolutionary features of the needle arthroscopy in ankle surgery [3, 4, 4, 7, 15, 17], the poorer image resolution compared to a standard arthroscope, and most importantly, the absence of distal obliquity and the 120° field of view made the lateral ankle ligament complex anatomical reconstruction using the needle arthroscopy-only approach, impossible in all three cases. The needle arthroscopy has as its main purpose to be an alternative to MRI imaging and second-look arthroscopy. However, this less invasive advent still presents crucial limitations in ankle ligament reconstruction in its classic arthroscopic form which, in this study, could not be performed using exclusively the needle arthroscopy. Although the small diameter and flexibility of the new tool seemed to be particularly well suited to the ankle joint, the different views obtained with the needle arthroscope, under the usual technical conditions (patient position and identical approaches), did not allow the completion of the surgical procedure. Exploration of the medial and lateral gutters by the anteromedial portal was always complicated, if not impossible, with the needle arthroscope. On the other hand, the evaluation of syndesmosis was always easier. The anterior chamber was also easily visualized with both tools.

An anatomical study had previously shown the interest of a different obliquity (70° optics) to better visualize certain areas of the ankle, particularly the very anterior and posterior part of the articular surface of the distal tibia [22]. A recent cadaveric study [18] confirmed these data with an exploration allowing in all cases (n = 10) the visibility of all anatomical elements (deep fibers of the medial collateral ligament, anterior bundle of the lateral collateral ligament, medial, lateral and anterior gutter, the entire talus) by standard anteromedial or anterolateral approaches as described by previous authors [6, 10]. Additionally, Stornebrink et al. [18] reported that it was possible to see an average of 96% of the talus dome and 85% of the articular surface of the distal tibia. Accordingly, in the present study, the articular surfaces were largely visualized, and we were able to move from the anterior to the posterior joint chamber without any difficulty. This feature seems to us to be particularly interesting in the context of the endoscopic treatment of osteochondral lesions because of the technical difficulties known to be related to reduced accessibility. Needle arthroscopy is also an option as the initial step in the management of a suspected joint reported 11 cases of bacterial arthritis of native joints (ankle, wrist, shoulder and knee) and needle arthroscopic led to successful lavage in all cases, requiring no further surgical interventions [19].

Iatrogenicity was also evaluated in a previous cadaveric study using the needle arthroscopy, and only in one case was an intermediate dorsal cutaneous branch of the superficial fibular nerve in contact with the anterolateral approach without macroscopic lesions [18]. The average distance between this nerve and this approach was 2.2 mm. The average distance between the anterolateral approach and the anterior vasculonervous pedicle was 8.8 mm [18]. The same authors performed the same cadaveric study evaluating fibular, posterior tibial, and calcaneal tendinoscopies and found similar results [20].

Although superficial nerve iatrogenic injury has a lower rate when the approach is limited to 2.2 mm, it cannot be eliminated because anatomical variability in the distal branches of the superficial fibular nerve is significant and unpredictable [8, 9, 21]. Contrary to what was reported by Stornebrink [18], it has been shown that transillumination does not reliably limit nerve damage in ankle arthroscopy [12]. To limit this superficial nerve damage, the position of the ankle in dorsiflexion seems to be important [6, 9], but ultrasound identification is reported to be the best solution [1, 2, 16]. No cartilage iatrogenic injury was reported in this same study [18], compared with the 31% found by Vega et al. [23]. The semirigid nature and the small diameter seemed to be major advantages in limiting cartilage damage.

**Fig. 7:**
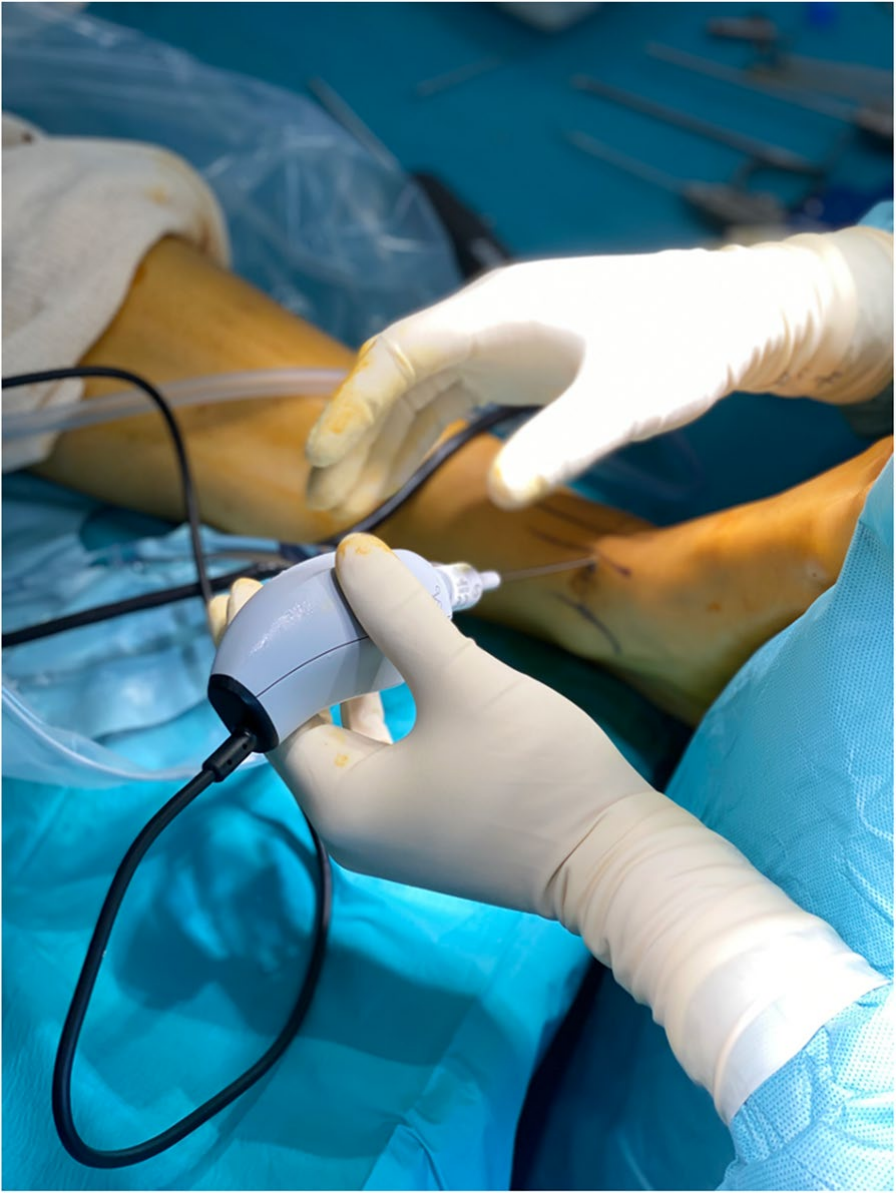
Needle arthroscope ergonomics

Finally, another important factor for ankle arthroscopy is ergonomics. The needle arthroscope is four times lighter than a standard arthroscope (Table 2), making its management easier, however stabilization in the three spatial planes is much more difficult (Fig. 7). Additionally, a learning curve is necessary to master the gestures particular to this camera.

The limitations of this study are mainly the fact that a single surgeon performed the all the procedures, and the small number of cases.

In conclusion, despite the innovative evolutionary features of the needle arthroscopy in ankle surgery, the poorer image resolution compared to a standard arthroscope, and most importantly, the absence of distal obliquity and the 120° field of view made the anatomical reconstruction of the lateral ankle, using the needle arthroscopy-only approach, impossible in all three cases.

The needle arthroscope should not be considered as a "mini arthroscope" but as a new tool with which it will probably be necessary to rethink and describe truly new surgical procedures to take advantage of the benefits of this instrument.
